# Novel *HRPT2/CDC73* Gene Mutations and Loss of Expression of Parafibromin in Chinese Patients with Clinically Sporadic Parathyroid Carcinomas

**DOI:** 10.1371/journal.pone.0045567

**Published:** 2012-09-20

**Authors:** Ou Wang, Chunyan Wang, Min Nie, Quancai Cui, Heng Guan, Yan Jiang, Mei Li, Weibo Xia, Xunwu Meng, Xiaoping Xing

**Affiliations:** 1 Key laboratory of Endocrinology, Ministry of Health; Department of Endocrinology, Peking Union Medical College Hospital, Peking Union Medical College, Chinese Academy of Medical Sciences, Beijing, People’s Republic of China; 2 Department of Pathology, Peking Union Medical College Hospital, Peking Union Medical College, Chinese Academy of Medical Sciences, Beijing, People’s Republic of China; 3 Department of Surgery, Peking Union Medical College Hospital, Chinese Academy of Medical Science & Peking Union Medical College, Beijing, People’s Republic of China; University of Porto, Portugal

## Abstract

**Objective:**

It is widely recognized that the diagnosis of parathyroid carcinoma (PC) is often difficult because of the overlap of characteristics between malignant and benign parathyroid tumors, especially at an early stage. Based on the identification of tumor suppressor gene *HRPT2/CDC73* and its association with hereditary and sporadic PC, screening of gene mutations and detection of parafibromin immunoreactivity have been suggested as diagnostic instruments of PC in Whites. There is little information about *HRPT2/CDC73* mutations and its corresponding protein expression in patients with sporadic PC in Chinese population, and the long-term follow-up data is scarce.

**Methods:**

Paraffin-embedded tissues were obtained from 13 patients with PC, 13 patients with parathyroid adenoma (PA) and 7 patients with parathyroid hyperplasia(PH), and 6 normal parathyroid (NP) tissues as controls. Peripheral blood from 11 patients with PC was collected. PCR products using Genomic DNA extracted from tumor tissues or blood as template was sequenced for *HRPT2/CDC73* gene. Expression of parafibromin in tumor tissues was evaluated by immunohistochemical analysis.

**Results:**

Six mutations in 6 of 13 patients with PC were identified, with three being novel. Four of them were germ-line mutations. Patients with mutations were susceptible to recurrence of the PC. Complete (8/13, 61.5%) or partial (5/13, 38.5%) loss of parafibromin expression was observed in PC tissues. All of tissue samples from normal parathyroid or benign parathyroid tumors displayed positive immunostaining of parafibromin except one adenoma.

**Conclusions:**

The present study supplies information on the mutations and protein expression of *HRPT2/CDC73* gene and phenotypes of parathyroid carcinoma in Chinese population. And the expanded mutation database of this gene may benefit patients in the diagnosis and treatment of this disease.

## Introduction

Sporadic primary hyperparathyroidism (PHPT) is mostly caused by parathyroid adenoma in approximately 80–85% of cases or parathyroid hyperplasia in 10–15% of cases. Parathyroid carcinoma (PC) is the uncommon but potentially life-threatening type, which accounts for less than 1% of PHPT cases in most western countries and about 5–7% of the PHPT cases in China, respectively [1]–[3]. The prevalence of PC was reported to be 5% in a nationwide study in Japan and 5.2% in a retrospective study in Italy, which was similar to that in China [4], [5]. Most cases of PHPT can be cured by parathyroidectomy while PC is one of the main causes of recurrent or persistent PHPT. *En bloc* resection at the initial surgery appears to improve the prognosis of PC. However, the distinction between parathyroid carcinoma and adenoma still often presents a diagnostic challenge at the early stage of PC in the absence of obvious metastasis [6], [7]. A definitive histopathological diagnosis of PC should be restricted to lesions showing vascular invasion, perineural space invasion, capsular penetration with growth into adjacent structures, and/or documented metastases [6]. It is widely recognized that making the diagnosis of PC is often difficult because of the overlap of characteristics between PC and parathyroid adenoma (PA), especially at an early stage.

Most of the recent genetic studies of PC have been based on the identification of the *HRPT2/CDC73* gene in chromosome 1q24–q32 and its corresponding protein parafibromin in 2002 [8]. Germ-line inactivation mutations of the tumor suppressor gene - *HRPT2/CDC73* are related to hyperparathyroidism-jaw tumor syndrome (HPT-JT, OMIM 145001), in which about 90% of cases presenting PHPT and up to 15% of cases presenting PC [9]. Somatic and germ-line *HRPT2/CDC73* mutations were also commonly found in about 67–100% of sporadic parathyroid carcinomas reported by Shattuck TM (10/15), Howell VM (4/4) and Cetani F (6/7) [10]–[12], but its mutations were found in only 3 among167 cases of parathyroid adenoma [13]. Parafibromin, the product of the *HRPT2/CDC73* gene, is the human homolog of the yeast Cdc73 protein which is a component of the Protein Associated Factor1 complex and involved in transcriptional and post-transcriptional control pathways [8], [14], [15]. Immunohistochemical staining of parafibromin suggested reduced or absent expression of parafibromin in sporadic PC tissue, but retained in most of the benign lesions of parathyroid glands [16]–[18]. Given the strong evidence linking inactivation of *HRPT2/CDC73* gene and malignant parathyroid tumor, gene mutations screening and detection of parafibromin immunoreactivity have been suggested as diagnostic instruments of PC.

To date, there are more than 110 germ-line and somatic mutations reported in *HRPT2/CDC73* gene, most of which are described in Whites [19]. Among them, thirty-five mutations were found in sporadic parathyroid carcinomas. In this study, we analyzed a series of Chinese patients with clinically non-familial parathyroid carcinoma in an effort to investigate the *HRPT2/CDC73* gene mutations and the different immunohistochemical expressions of parafibromin in the PC tissue compared with that in adenoma, hyperplasia and normal tissue of parathyroid glands.

## Materials and Methods

### Patients and Tissue Samples

From 1986 to 2006, 20 patients were diagnosed as PC according to clinical, radiographic, surgical, and histological features in Peking Union Medical College Hospital. Among them, paraffin-embedded tissues from 13 patients were available for genomic DNA extraction and most of the patients had been followed up for at least 3 years. Paraffin-embedded tissues from 13 patients with sporadic parathyroid adenoma (PA), 7 patients with sporadic parathyroid hyperplasia (PH) during the same period were used as controls. They were age- and gender-matched to the PC cases. Six normal parathyroid (NP) tissues from patients undergoing subtotal thyroidectomy for hyperthyroidism or thyroid goiters were used as normal controls. No patient had a history of irradiation to the head and neck or renal dysfunction. None of these patients had a family history of hyperparathyroidism. Since most of the family members of the index patients refused genetic analysis, the first-degree relatives were suggested to have their serum calcium and PTH levels tested to reduce the probability of familial hyperparathyroidism. Eleven blood samples of the 13 patients with PC were collected into EDTA anticoagulant tubes and stored at −80°C.

### Ethics Statement

The present study was approved by the local Ethics Committee of Peking Union Medical College Hospital. The written informed content was obtained from all subjects.

### Mutation Screening of the *HRPT2/CDC73* Gene

Genomic DNA was extracted from paraffin-embedded tumor tissue and peripheral blood leukocytes using QIAGEN DNA extraction kit (QIAGEN company, Germany). Coding sequence abnormalities in the *HRPT2/CDC73* gene were assessed by PCR and then direct sequencing analysis. The complete coding region including all exons and intron-exon boundary was amplified with the primers previously described [20] or designed by Primer 5.0 (sequences listed in Table 1). All PCRs included 100–300 ng of genomic DNA template, 20 pmol of each primer, 20 µl of 2×easytaq mix (Tiangen Biotech, Beijing, China) and double-distilled water to obtain a final volume of 40 µl in a gradient thermocycler (MJ Research PTC-200, Bio-Rad, USA). Amplifications were carried out as follows: an initial denaturation for 2 min at 94°C followed by 35 cycles of 94°C for 30 s, 49–65°C for 30 s, 72°C for 30 s, and a final extension for 8 min at 72°C. Direct sequencing of PCR products was performed using a Taq big dye terminator sequencing kit and an ABI3730 automated sequencer (Applied Biosystems, Foster City, CA, USA). Sequencing was performed in duplicate. Both sense and anti-sense strands were sequenced. Sequences generated from patients were compared with the published *HRPT2/CDC73* sequence (NCBI reference sequence: NG_012691.1). All of the mutations were confirmed by suboclonal sequencing.

**Table 1:** The primer for amplification of *HRPT2/CDC73* gene.

Exon	Forward primer	Reverse primer	Length (bp)	Temperature(°C)
1	CGAGGCGACAAGAGAAGAAG	ACACCCGTTTTATCCCATCC	324	60.5
2	TCCAGCCTGAAGAGTTGAAT	GATCACACCACTGCACCCTA	275	55.0
3	TACAAATGTGATTTAAAATACG	TGTATA AGAAAAAGGTGAGCAC	350	49.0
4	CCTAAAGCATTTCACTTGTAA	GTGTAGTTTTGGAATGGG	199	53.0
5	GAACTTTCAGAAGCCCATTC	TGAGCCAATAGGTTCATCCA	361	59.1
6	TGAAGTTGGCCTAAAGACAC	GACTTCCAATCCCCACACATG	385	62.5
7	ACTCCAGGAATGCCTGCTGTG	CCACAGATTAAACGCACTCTC	332	64.9
8	TAGTAGGGAAGAATCGATAG	CTTCAACGTTACTACACTGC	276	60.4
9	AGGAGGTCTTGGCTGCAGTG	GTAAGATCTGGTCTGTAGAC	304	62.9
10	ACAATAGGCTTGCTGGTCTG	TCCCTGGAACAAAAGAACATC	332	59.0
11	CAGTGGAGTAACCAACTGAGTGA	GCTGACTGAAGTTTAGCAAGCA	395	59.8
12	GGACTGTGGTTAACTGAAACTGC	CAGGCCTGGTTTTGCAATCA	361	64.3
13	GCCCAAGCCACACTGATTAT	AAGGCCTATAGCACAGAAACCG	375	61.8
14	ATCTTCCCATTTTCATCA	GGGCTGGTCTACTACTCT	199	52.9
15	GAAATAATCTCCGTCTGTCCCC	CACATCATATGCGCAGAACT	246	61.0
16	ATACGGCTTCAGTTGGTGGA	GACTGACAACTCTATGGAAC	318	60.5
17	CAGGTACATGGTAAAGCATA	TGCTAATTTAACCAATGGAG	294	53.1

Exon 2, 3, 4, 8 and 14 were designed by Primer5.0, the others as reference [^18^].

### Parafibromin Immunohistochemistry

For immunohistochemistry, sections were obtained from paraffin-embedded tissue blocks. Experiments were performed as previously described to investigate the expression of parafibromin in different type of tumors by immunohistochemistry [21]. Briefly, serial sections from formalin-fixed, paraffin-embedded tissues were collected onto poly-L-lysine–coated slides, and 4-µm sections were used for immunostaining. Archival sections were deparaffinized in xylene and rehydrated in alcohol. Endogenous peroxide activity was blocked by incubating the slides in 3% hydrogen peroxide in dehydrated alcohol for 10 min. After a heat-induced antigen retrieval procedure in 10 mM citrate buffer (pH = 6.0) for 30 min, in order to unmask the antigen, slides were cooled at room temperature for 30 min and then blocked against nonspecific staining with 1% BSA serum. Primary antibodies against parafibromin (Santa Cruz, USA; 1∶40) were incubated with the specimens for 18 h at 4°C. The slides were then washed with PBS (pH 7.2–7.4) and incubated with horseradish peroxidase-labeled secondary antibody for 1 h at 37°C. Liquid 3,3′ diaminobenzidine (DAB)+ chromogen was added and incubated for another 6 min. Finally, sections were counterstained with hematoxylin, dehydrated, and mounted. The positive control for parafibromin was normal parathyroid tissue. Omission of the primary antibody and replacement with 0.01 mM PBS was included as a negative control.

Cells were scored as positive if specific nuclear staining was detected, and staining was quantified according to the percentage of positive cells independent of the intensity of staining. For parafibromin, no reactivity was scored as (–), percentage of positive cells <50% as (+), percentage of positive cells 50∼95% as (++), and percentage of positive cells >95% as (+++). Each section was evaluated by two pathologists (Dr. DZ and Dr. JS) without knowledge of the diagnosis or outcome. Images were acquired using an Olympus Bx51 microscope (Olympus Corp, Japan) with a mechanical stage, fitted with an Olympus u-CMAD3 videocamera (Olympus Corp, Japan); the latter was connected to a Pentium II computer located with the appropriate image analysis software. Slides were examined at high power magnification (original magnification,×200).

### Statistical Analysis

The statistical software package SPSS for Windows version 10.0 (SPSS, Chicago, IL, USA) was used to analyze the data. The normally distributed data was summarized by the mean±SD and comparisons between groups were made by one-way ANOVA. The non-normally distributed data was summarized by the median and range. The chi-square or Fisher’s exact test was performed to compare the status of mutation and parafibromin expression between subjects with PC and benign parathyroid tumors. The binary losgistic regression was used to adjust for the baseline covariates. Sensitivity, specificity, and predictive values were calculated using standard methods for binomial distribution.Differences were considered statistically significant at *p*<0.05.

## Results

The clinical characteristics of the 13 patients of PC were summarized in Table 2. There were 10 males and 3 females with a median of history of PHPT being 1.00 year (ranged from 0.08∼13.00 years). The mean age at the diagnosis of PHPT and PC were 48.1±14.6 and 50.2±14.2 yrs, respectively. Three patients (Ca2, Ca8, Ca11) were diagnosed with PA at the initial surgery but PC after the second surgery. The size of parathyroid lesions at the first surgery was 3.44±1.33 cm. The serum calcium was 3.84±0.58 mmol/L and PTH levels were 22.36±10.22 folds of upper limit of normal range before the initial parathyroidectomy. Nine of the thirteen (69.2%) patients were admitted for hypercalcemic crisis (defined by serum calcium ≥3.5 mmol/L) before the first surgery. The kidney cystic lesions were observed in only 2 patients detected by abdominal ultrasound or CT scanning and only 1 patient was suspected to have jaw tumor suggested by CT scanning. However, further biopsy was not accepted by the patient. No uterine tumors were detected by pelvic ultrasound among the female patients. The serum calcium and PTH levels of the first-degree relatives of the index were normal. None of the patients was diagnosed as PC before surgery so that only parathyroid tumors were resected at the initial operation. Among patients followed-up for 6.6±5.1 years (ranged from 0.25–14 years), distal metastasis was proved in 4/12 patients. Eight patients experienced at least one recurrence of hypercalcemia among the 10 patients followed for at least 1.5 year and the first recurrence was detected in 3.4±2.2 years (1.0∼7.0 years) after the first surgery. Eight patients were re-operated at least twice with a range of 2–10 times. Patient Ca6 and Ca10 died of intractable hypercalcemic crisis 10 and 14 years after the first parathyroidectomy, respectively.

**Table 2:** Clinical characteristics and *HRPT2/CDC73* mutations in patients of parathyroid carcinoma.

Case no.	Gender	Historyof PHPT(year)	Age at diagnosis of PHPT/PC	#Size of tumor (cm)	*Serum Ca (mmol/L)	*PlasmaiCa(mmol/L)	*SerumP(mmol/L)	*SerumALP(U/L)	*Serum PTH(×UL)	Follow-upafter firstsurgery(year)	Distant metastasis	Recurrence	Kidneycysticlesions	Jawtumor	*HRPT2*mutation	Parafibrominstaining
ca1	Male	0.08	50/50	3.5	4.78	2.80	0.65	304	30.0	3.17	No	No	No	No	No	++
ca2	Male	1	36/45	NK	NK	NK	NK	NK	NK	11.00	Lung	Yes	NK	NK	No	–
ca3	Male	0.67	39/39	3.0	2.96	NK	0.74	1676	17.4	0.33	No	–	NK	NK	No	–
ca4	Female	0.5	71/71	2.5	3.50	1.71	NK	115	14.9	–	–	–	NK	NK	No	–
ca5	Male	0.25	58/58	1.5	4.66	2.06	0.33	91	17.1	3.25	No	Yes	No	No	Yes	+
ca6	Male	1	29/29	3.0	4.03	2.05	0.55	530	14.9	14.00	No	Yes	Yes	Yes	Yes	–
ca7	Male	3	41/41	3.5	4.28	2.31	0.87	945	47.2	0.25	No	–	No	No	No	–
ca8	Male	13	55/67	6.0	3.08	1.52	0.42	1087	16.8	13.00	No	Yes	No	No	Yes	+
ca9	Female	11	69/69	5.0	3.30	NK	0.55	NK	28.4	6.00	No	No	No	No	No	–
ca10	Male	1	40/40	NK	3.67	NK	NK	NK	NK	10.00	Lung	Yes	No	No	Yes	–
ca11	Female	0.25	24/30	3.0	3.85	NK	0.73	392	16.9	11.50	No	Yes	No	No	No	–
ca12	Male	0.17	60/60	NK	3.80	NK	0.50	662	20.0	1.67	Lung	Yes	Yes	No	Yes	+
ca13	Male	1	53/53	NK	4.13	NK	0.77	NK	NK	4.70	Lung, skeleton	Yes	No	No	Yes	+

# size of the parathyroid tumor at the first surgery;

* biochemical markers before the first surgery; NK: not known; UL: upper limit.

The clinical characteristics of subjects in control groups were summarized in Table 3. There were no significant difference in the male/female ratio, history of PHPT (4.1±4.6 years, ranged from 0.5∼20 years), age at diagnosis of PHPT (49.5±13.7 years), size of tumors (2.97±1.18 cm), and biochemical markers including serum calcium (3.18±0.40 mmol/L), serum PTH (17.3±13.8 folds of upper limit of normal range) of patients with benign parathyroid tumors compared to those with PC.

**Table 3:** Clinical characteristics and *HRPT2/CDC73* mutations in subjects of control groups.

Case no.	Gender	History of PHPT(year)	Age at diagnosis of PHPT(year)	Size of tumor (cm)	Serum Ca (mmol/L)	Plasma iCa (mmol/L)	Serum P (mmol/L)	Serum ALP (U/L)	Serum PTH (×UL)
pa1	Male	0.5	50	4.5	3.02	1.55	0.65	112	8.5
pa2	Male	3	36	4	2.88	NK	0.55	2013	33.6
pa3	Male	0.17	38	1.5	3.05	1.46	0.81	54	4.4
pa4	Female	6	69	1.7	2.93	1.37	0.81	99	4.1
pa5	Male	20	52	2.5	3.03	1.49	0.68	72	6.4
pa6	Male	0.5	30	4	3.10	1.67	0.52	749	21.5
pa7	Male	1	42	3	3.40	1.76	0.81	109	7.4
pa8	Male	3	63	2.5	3.51	1.52	0.84	145	6.4
pa9	Female	1	63	2.5	3.51	1.40	0.83	146	31.5
pa10	Male	2	40	5	4.48	2.21	0.97	323	34.5
pa11	Female	6	41	5	3.10	1.73	0.65	1833	47.2
pa12	Male	10	60	3	3.15	1.62	0.81	1530	35.8
pa13	Male	8	54	1.8	2.86	1.34	0.96	105	6.8
ph1	Male	4	57	3	2.60	NK	0.94	103	3.7
ph2	Female	2	76	2	3.48	NK	0.61	85	6.6
ph3	Male	0.75	28	2.5	3.35	NK	0.61	80	10.3
ph4	Male	2	45	4	3.63	NK	0.45	178	31.2
ph5	Male	2	42	1.2	2.87	NK	0.39	NK	4.0
ph6	Female	6	67	2	2.90	1.53	0.90	200	20.0
ph7	Male	5	37	4	3.13	NK	0.77	154	21.5
NP1	Male	–	58	–	2.16	–	1.15	–	–
NP2	Male	–	46	–	2.21	–	1.41	–	–
NP3	Female	–	52	–	2.38	–	1.34	–	–
NP4	Female	–	52	–	2.36	–	1.14	–	–
NP5	Male	–	60	–	2.25	–	1.10	–	–
NP6	Female	–	50	–	2.28	–	1.13	–	–

pa: parathyroid adenoma; ph: parathyroid hyperplasia; NP: normal parathyroid; NK: not known; UL: upper limit.

### Genetic Analysis

**Figure 1:**
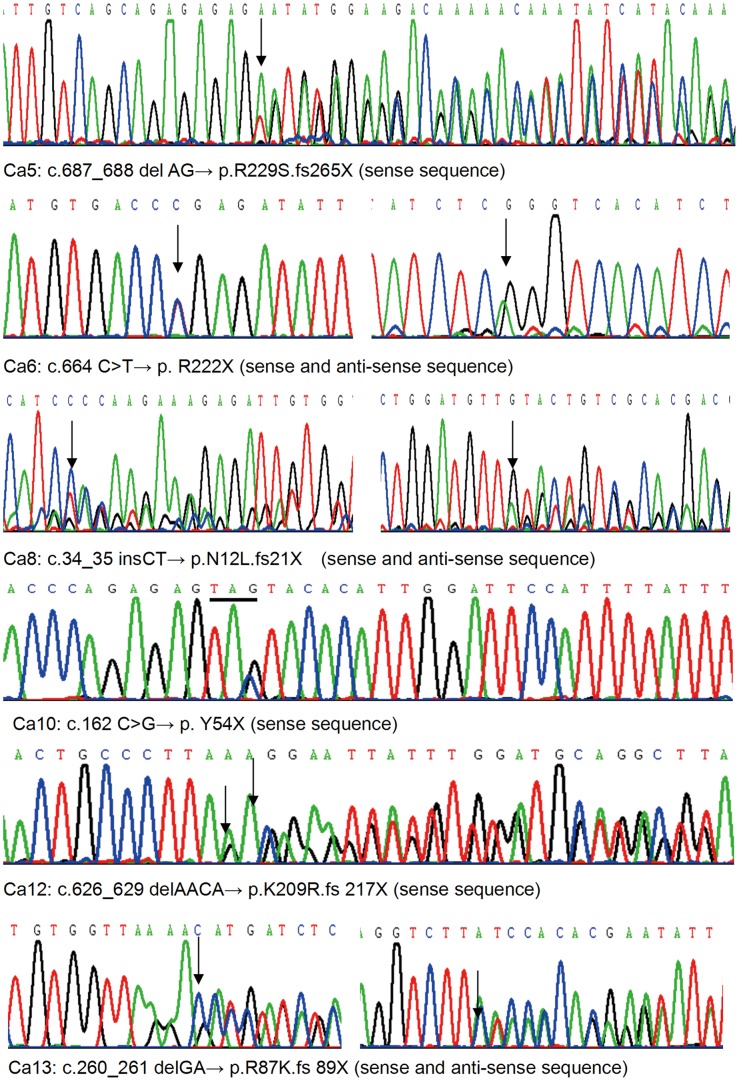
Mutations of *HRPT2/CDC73* gene.

Direct sequencing of the coding regions of *HRPT2/CDC73* gene identified 6 different mutations in 6 out of 13 paraffin-embedded tissues of PC (Table 2 and 4, Figure 1). Four of them were identified to be germline mutations by sequencing the PCR products amplified using the genomic DNA extracted from peripheral blood lymphocytes as template. The source of the other two mutations could not be determined because of lacking the peripheral blood. The frame shift mutation in Ca5 had been reported previously in HPT-JT syndrome and missense mutation in Ca6, frame shift mutation in Ca10 had been reported in sporadic parathyroid carcinoma. Three frame shift mutations causing truncated protein in Ca8, Ca12 and Ca13 were novel. No mutations were detected in any of the PA and PH tumors as controls.

**Table 4:** Mutations of HRPT2/CDC73 gene in patients with parathyroid carcinoma.

Case no	Nucleotide alteration	Exon	Mutation type	Predicted effect	Reference
Ca5	c.687_688 delAG p.R229S.fs 265X	7	Germline	Frame shift/premature stop codon	Howell et al., 2003[^9^]
Ca6	c.664 C>T p. R222X	7	Germline	Missense mutation/premature stop codon	Shattuck et al., 2003[^8^]
Ca8	c.34_35 insCT p.N12L.fs21X	1	ND	Frame shift/premature stop codon	Novel
Ca10	c.162 C>G p. Y54X	2	ND	Missense mutation/premature stop codon	Howell et al., 2003[^9^]
Ca12	c.626_629 delAACA p.K209R.fs217X	7	Germline	Frame shift/premature stop codon	Novel
Ca13	c.260_261 delGA p.R87K.fs 89X	3	Germline	Frame shift/premature stop codon	Novel

ND: not determined owing to the unavailability of germ-line DNA.

The patients with PC were further classified into two subgroups according to the status of *HRPT2/CDC73* gene mutation. There was no significant difference in the gender, age at diagnosis of PHPT/PC, history of PHPT, tumor size, distant metastasis and biochemical markers between the patients with and without mutation (Table 5). Among the patients followed for more than 1.5 year, the incidence of recurrence was significantly higher in patients with mutation than in those without mutation (100% *vs.* 50%, p = 0.031). After adjusting for gender, history of PHPT, age, initial serum calcium and the duration of follow-up by binary logistic regression, the difference was still significant (p = 0.023).

**Table 5:** Comparison between the PC patients with and without HRPT2/CDC73 mutation.

	With mutation	No mutation	P value
n	6	7	
Gender (M/F)	6/0	4/3	0.122
History of PHPT(years)	2.7±5.0	2.4±3.9	0.882
Age at diagnosis ofPHPT (years)	49.2±12.1	47.1±17.4	0.816
Age at diagnosis of PC (years)	51.2±14.1	49.3±15.4	0.824
#Size of parathyroid lesion (cm)	3.50±2.29	3.42±0.86	0.936
*Serum Ca (mmol/L)	3.90±0.52	3.78±0.67	0.744
*Serum P (mmol/L)	0.51±0.16	0.71±0.12	0.066
*Serum PTH (×UL)	17.2±2.1	25.8±12.3	0.210
Metastasis (Yes/No)	3/3	1/5	0.296
Recurrence (Yes/No)	2/2	0/6	0.031

# size of the parathyroid tumor at the first surgery;

* biochemical markers before the first surgery; NK: not known; UL: upper limit.

### Immunohistochemical Analysis

**Figure 2:**
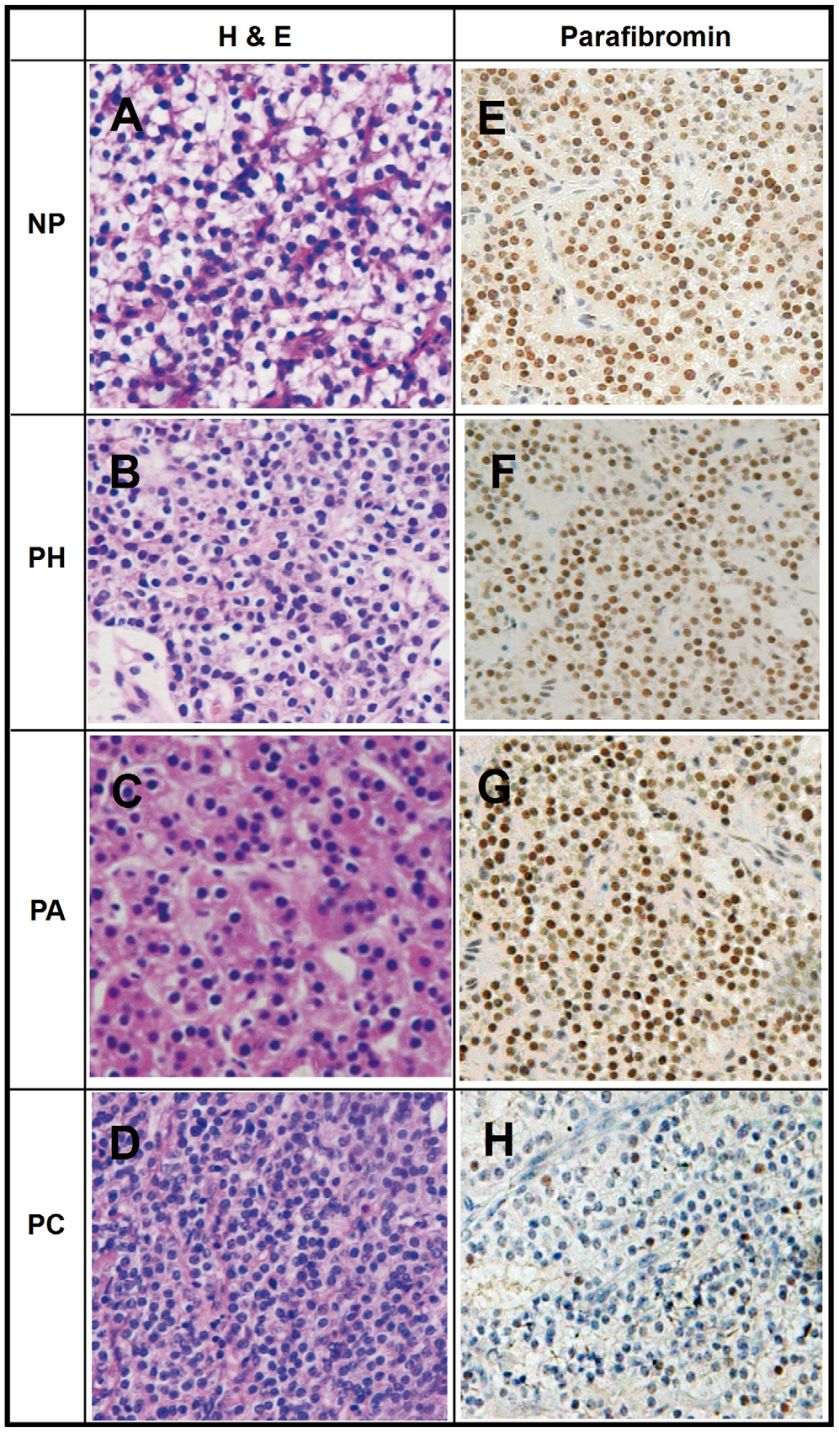
H&E staining and IHC staining in tissue samples of different types of PHPT(200×). NP, normal parathyroid tissue; PH, parathyroid hyperplasia PA, parathyroid adenoma; PC, parathyroid carcinoma A–D H&E staining of normal parathyroid tissue,parathyroid hyperplasia, parathyroid adenoma and parathyroid carcinoma E–G IHC of Parafibromin is specific nuclear staining in normal parathyroid tissue,parathyroid hyperplasia, parathyroid adenoma, brown granulation can be seen in positive nuclear side H Typical loss staining of Parafibromin in PCA–D H&E staining of normal parathyroid tissue,parathyroid hyperplasia, parathyroid adenoma and parathyroid carcinoma E–G IHC of Parafibromin is specific nuclear staining in normal parathyroid tissue, parathyroid hyperplasia, parathyroid adenoma, brown granulation can be seen in positive nuclear side H Typical loss staining of Parafibromin in PC.

As parathyroid glands expresses high levels of parafibromin and shows strong nuclear immunostaining, normal parathyroid tissues, parathyroid hyperplasia and adenomas were used as positive controls (Figure 2). The immunoreactivity of all NP tissues and benign parathyroid tumors (including PA and PH) was scored as (+++) except of one PA scored as (−). Complete loss of parafibromin expression was observed in 8 of 13(61.5%) PC tissues and partial loss (scored +∼++) of parafibromin expression in the other 5 of 13 PC tissues as shown in Table 2. The odds ratio of complete loss of parafibromin expression for diagnosis of PC was 2.470 (95% confidence interval: 1.233∼4.949, *p* = 0.002).

### Utility in the Differential Diagnosis of Malignant and Benign Parathyroid Tumors

The mutations of *HRPT2/CDC73* gene were detected only in PC tissues with a sensitivity of 46.2% and specificity of 100% for diagnosis of PC. The sensitivity and specificity of complete loss of parafibromin expression in diagnosis of PC was 61.5% and 95%, respectively. Combination of above two markers-mutations in *HRPT2* and complete loss of parafibromin expression increased the sensitivity to 92.3% without reduction in specificity.

## Discussion

The different incidence of parathyroid carcinoma in all cases of PHPT between most western countries (<1%) and China (5–7%) suggested a regional variation. The disparity might be caused by several reasons including genetic variation or the difference in the severity of PHPT. With the widespread screening of serum calcium levels, most of the patients of PHPT are asymptomatic in western countries. While in China, the majority of the patients are sympotomatic with bone resorption in more than 70% and nephrolithiasis in more than 40% of cases [3]. Of note, the limited number of cases of PHPT reported in China may lead to biased data, so the larger and nationwide surveys are needed. To our knowledge, this is the first systemic study on the role of *HRPT2/CDC73* gene mutation and its protein expression in a series of Chinese patients with clinically non-familial PC with the long-term follow-up for up to 14 years. And the sample size of the present study was comparable to the congeneric studies on the role of *HRPT2/CDC73* gene mutation in sporadic PC from single center.

In this series of patients with PC, males accounted for 76.9% of the cases and were more common than in case series from Caucasian populations (approximately 50%). PC is reported to occur about 10 years earlier than benign PHPT. However, the mean age at the diagnosis of PHPT/PC in this group was 48.1/50.2 years, which was similar to patients with PA or PH in our hospital [3]. Recently, the mean age at the diagnosis of PC pooled from published data by Sharrets JM et al was 44–48 years with a range of 12–90 years, which is similar to our results [22]. Similar to previous studies, the laboratory tests displayed significant elevation of serum calcium and PTH levels in this series of patients with PC, with a calcium level greater than 3.5 mmol/L in 69.2% of the cases [7]. Due to the rarity and difficulty in diagnosis before the surgery, no patients in the present study were operated by *en bloc* resection. 80% of the patients developed recurrence 1 to 7 years after the initial surgery and approximately 30% developed distant metastasis.

In the present study, we identified six *HRPT2/CDC73* mutations in 46.2% of cases with PC and none of those with benign parathyroid tumors. The prevalence of mutation in PC cases in this study is relative lower than that in the previous studies reported by Shattuck TM (10/15), Howell VM (4/4) and Cetani F (6/7) [10]–[12], indicating a race difference. The difference in the inclusion criteria might also accounted for the variation. The diagnosis of PC in early studies was more strict and most of the cases developed distal metastasis and/or recurrence. The percentage of metastasis or recurrence was 86.7% (13/15) in Shuttuck’s study compared to 61.5% (8/13) in our study. In the present study, four of the six mutations were certified to be germ-line mutations. Since renal cysts were found in two patients carrying germ-line mutations and jaw tumor was suspected in one of them, HPT-JT syndrome should be diagnosed in them even without family history. Then there were at least 18% of patients carrying germ-line mutations among the other eleven cases with sporadic PC. Shattuck TM has also reported that about 20% of patients with apparently sporadic PC carry germ-line *HRPT2/CDC73* mutations [10]. It is indicated that detection of the germ-line mutations in patients with sporadic PHPT may profit the diagnosis of PC before surgery. Recently, Witteveen JE reported an association between *HRPT2/CDC73* mutations and sevenfold increased risk of developing local or distant metastasis. All the four patients with gene mutation developed local recurrence and/or distant metastasis, which occurred in only 10 of the 19 patients without mutations (log-rank test: p = 0.08) [23]. Different from that, we did not find significant correlation between gene mutation and distal metastasis (50% in patients with mutation *vs.* 16.7% in those without mutation, p = 0.273) in this study. However, the higher incidence of recurrence in patients with mutations suggested the necessity of more intense and long-term monitoring in this subgroup. Due to the limitation in the sample size of the present study, the association between *HRPT2/CDC73* mutations and recurrence or metastasis of PC should be further studied in more samples.

The findings of germline mutations of *HRPT2/CDC73* gene in the previous and current studies are supportive to its role in the tumorogenesis [10]–[12]. On the other hand, it has been reported that some patients with apparently sporadic PC harbouring *HRPT2/CDC73* mutation turned out to have HPT-JT syndrome or phenotypic variants of the syndrome with altered penetrance on further investigation [10]. Therefore, genetic screening of the family members of the index patients for early detection or prevention of parathyroid malignancy is indicated, especially in patients with features of HPT-JT syndrome. It is regrettable that most of the family members in the current study did not accept the genetic analysis so that the parathyroid involvement in them were only exluded biochemically. The long-term surveillance of parathyroid, renal, jaw and uterine neoplasia of both the index cases and their family members is recommended.

Three of the six mutations in this study located in exon7 (50%) and the other three in exon1, 2 and 3, respectively. It is suggested that mutations in exon7 is more common in this cohort of Chinese patients. Newey PJ *et al* summarized 111 mutations occurred throughout the coding region and splice sites of this gene in patients with HPT-JT syndrome or parathyroid tumors and the three novel mutations in the present study represents 2.7% of them [19]. They found that mutations in exon1, 2 and 7 were overrepresented accounting for 34, 17 and 21%, respectively. The overrepresentation still remained even after adjusting for exon size. It is suggested that screening mutations in “hot” exons of *HRPT2/CDC73* gene may be cost-effective in suspecting patients with PC or HPT-JT syndrome.

In this study, all of the six mutations including three novel mutations resulted in a premature stop codon. Paralleling with it, immunohistochemical analysis also displayed complete loss or more than 50% reduced nuclear expression of parafibromin in the tumor with mutations. But the staining score was not correlated with the length of truncated protein. Interestingly, reduced or even absent parafibromin staining was also observed in PC tumors without a mutation. There may be some reasons for the discrepancy between the results of mutation screening and immunostaining of parafibromin. Firstly, we only detected the mutations in the coding region of the *HRPT2/CDC73* gene. The mutations harbored in introns and 5′- or 3′-UTR might be missed, which may affect the splice of the DNA or be involved in the regulatory processes of expression [24]. Secondly, epigenetic regulation may play a role in the loss of expression of parafibromin. Hewitt KM et al reported that methylation of the *HRPT2/CDC73* promoter had been detected in 2/11(18%) of PC but 0/37 of sporadic PA tumors [25] This result suggested epigenetic mechanisms including methylation of DNA, histone modifications, or RNAs interfering may be involved in the inactivation of *HRPT2/CDC73* gene [26]. Thirdly, the expression of parafibromin was evaluated by nuclear immunoreactivity of the protein, which might be not sensitive or specific enough. Hahn MA *et al* demonstrated three nucleolar localization signals of parafibromin at amino acid residues 76–92, 192–194 and 125–139, besides its nuclear localization signal at 125–139 [27]. It was shown that parafibromin is a nuclear protein in the co-localization studies with the nuclear marker. Recently, Juhlin CC et al assessed nuclear parafibromin immunoreactivity in different types of parathyroid tumors [28]. They observed the absent nucleolar expression of parafibromin in three carcinomas carrying *HRPT2/CDC73* inactivating mutations, which also showed expression of nuclear parafibromin in all or part of tumor cells, suggesting increased sensitivity and specificity for the detection of malignancy compared to scoring of nuclear parafibromin alone. Fourthly, there may be mutations in other genes which has not been recognized so far, which may affect the expression of parafibromin.

In the present study, we also assessed the value of *HRPT2/CDC73* mutation screening and immunohistochemical analysis of parafibromin in the differential diagnosis of malignant and benign parathyroid tumors. Similar to the results of previous studies in Caucasians [16]–[18], a specificity of 100% by mutation screening or immunostaining of the protein alone and a sensitivity up to 92.3% by combination of them together for diagnosis of PC were confirmed in this group of Chinese patients. Besides, the high percentage of germ-line mutations in this group makes it possible to obtain the diagnosis of PC before surgery. This may help the surgeon to choose optimal operative procedure to improve the prognosis of such patients. In a subgroup of patients with parathyroid atypical adenoma which is not definitely benign or malignant, the mutation testing and immunohistochemical analysis of parafibromin may be valuable. A positive expression of parafibromin would point toward benign disease, while detection of gene mutation and/or loss of parafibromin expression are suggestive of potential malignancy or HPT-JT syndrome. In the latter situation, intensified follow-up or further evaluation with other molecular markers (such as protein gene product 9.5, APC or galectin-3) are rational [29]. It would be more feasible to perform the genetic analysis in patients suspected of PC, atypical adenoma or HPT-JT syndrome, but not those with typical adenoma since benign tumors still account for more than 90% of PHPT in China population and such test is still not generally developed in China. Further studies in more subjects from multi-centers with more molecular markers are needed to work out the management algorithm of these patients in China.

In summary, we have firstly demonstrated the high prevalence of *HRPT2/CDC* mutations and loss of expression of its corresponding protein in clinically sporadic parathyroid carcinomas in Chinese population. Three novel *HRPT2/CDC* mutations have been identified. Genetic testing offers the possibility to diagnose not only hereditary but also sporadic parathyroid carcinoma at an early stage. Long-term and more closely follow-up is required in patients of parathyroid carcinoma for high rates of recurrence and distal metastasis, especially in *HRPT2/CDC73* mutation carriers.
